# Understanding the relative roles of local environmental, geo‐climatic and spatial factors for taxonomic, functional and phylogenetic *β*‐diversity of stream fishes in a large basin, Northeast China

**DOI:** 10.1002/ece3.9567

**Published:** 2022-12-12

**Authors:** Naicheng Wu, Yuanyuan Lv, Min Zhang, Yaochun Wang, Wenqi Peng, Xiaodong Qu

**Affiliations:** ^1^ Department of Geography and Spatial Information Techniques Ningbo University Ningbo China; ^2^ State Key Laboratory of Simulation and Regulation of Water Cycle in River Basin China Institute of Water Resources and Hydropower Research Beijing China; ^3^ Department of Water Ecology and Environment China Institute of Water Resources and Hydropower Research Beijing China

**Keywords:** biodiversity protection, ecosystem, metacommunity, nestedness, turnover, *β*‐diversity

## Abstract

The primary objective of this study was to determine the relative roles of local environmental (*Local*), geo‐climatic (*Geo*), and spatial (*Spatial*) factors to taxonomic, functional, and phylogenetic *β*‐diversity of stream fish in a large basin in Northeast China. We quantified the current biodiversity patterns of fish communities in the Hun‐Tai River using *β*‐diversity. We assessed (i) corresponding contributions of turnover and nestedness within the taxonomic, functional, and phylogenetic *β*‐diversity of fishes; (ii) correlations among *β*‐diversity facets (i.e., taxonomic, functional, and phylogenetic facets); (iii) relative contributions of *Local*, *Geo*, and *Spatial* factors to *β*‐diversity. We collected fish communities from 171 sampling sites. Mantel tests were used to examine the correlation of three facets of *β*‐diversity and their components (i.e., total, nestedness, and turnover). Distance‐based redundancy analysis and variation partitioning assess the relative contributions of *Local*, *Geo*, and *Spatial* factors to *β*‐diversity. We found that turnover is the main driving mechanism for *β*‐diversity in fish. Among the facets of *β*‐diversity, taxonomic and phylogenetic facets have strong ecological information association. *Spatial* factors have a general contribution to various facets of *β*‐diversity and its components. From aspects of fish *β*‐diversity conservation, connectivity and habitat heterogeneity need to be maintained in the entire aquatic environment. In addition, protecting taxonomic *β*‐diversity is helpful for maintaining phylogenetic *β*‐diversity.

## Graphical abstract

We found that turnover is the main driving mechanism for *β*‐diversity in fish, with more functional loss than functional turnover in fish assemblages; among the facets of *β*‐diversity, taxonomic and phylogenetic have strong ecological information association; spatial factors have a general contribution to various facets of *β*‐diversity and its components.

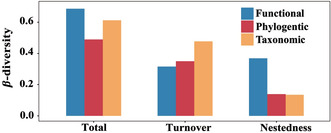

## 1 INTRODUCTION

Understanding the different facets of *β*‐diversity and its ecological drivers is essential for community ecology and freshwater conservation (Coleman et al., 2015; Devictor et al., 2010; Wu et al., 2022). By measuring biological *β*‐diversity, it is possible to prioritize its conservation (Appolloni et al., 2017; McKnight et al., 2007). *β*‐diversity can be decomposed into turnover and nestedness components to quantify the interregional volatility index (Baselga, 2010). Turnover is related to the displacement of species between regions and may be affected by dispersal limitations and environmental filtering (Baselga, 2010; Pelaez & Pavanelli, 2019). Nestedness shows that the community with less species composition is a subset of the community with rich species (Baselga, 2010; Soininen et al., 2018). In previous studies, changes in community composition were explored mainly based on taxonomy (Taylor, 2010; Villeger et al., 2015). Understanding the percentage differences in the species composition of different communities, and providing information on species overlap and differences, brings key conceptual advances in the patterns behind differences in species composition (Qian et al., 2021). However, it is universally accepted that taxonomy is not enough to understand species' structural and evolutionary information without considering the functional and phylogenetic facets of biodiversity (Gianuca et al., 2017; Taylor, 2010). In recent years, increased research on other facets of *β*‐diversity has allowed for a more complete understanding of the *β*‐diversity operating mechanism (Hill et al., 2019; Qian et al., 2021; Wu et al., 2022). First, species phylogeny can detect community similarity in cases where two communities do not share species. In other words, in the absence of common species between two communities, we can determine the linkages and affinities between species based on historical interspecific information by studying phylogenetic and taxonomic facets. This may promote our understanding of the historical evolution of communities and contemporary ecological changes (Cassia‐Silva et al., 2019; Qian et al., 2020). Second, by studying the functional traits of species, we can understand the structure of species combinations and their impact on ecosystems and help to understand the community aggregation process at different spatial scales and develop ecological conservation strategies (Coleman et al., 2015; Wang et al., 2018). Additionally, it can also provide a helpful perspective on the spatial and temporal variations of species distribution and abundance (Frimpong & Angermeier, 2009) and address contemporary issues in freshwater fish management and conservation. These three facets of *β*‐diversity help to elucidate the potential effects of environmental, spatial factors, and evolutionary processes on organisms and provide a deeper understanding of the dynamics of ecosystems at all levels (Goncalves et al., 2020; Heino & Tolonen, 2017).

Identifying the mechanisms that drive local environmental and intercommunity change is at the heart of community ecology (Baselga, 2010; Padial et al., 2014; Perez Rocha et al., 2018). Several previous studies have pointed out that local environmental (*Local*) variables are the main structural factors influencing fish communities (Araujo et al., 2019; Leprieur et al., 2012; Lopez‐Delgado et al., 2020). But, in recent years, more scholars have found that river biodiversity patterns are driven by a variety of abiotic factors (Goncalves et al., 2020; Grenouillet et al., 2008; Vardakas et al., 2020). Especially, spatial (*Spatial*) factors are important for organisms with relatively low dispersive capacity, such as aquatic fish and macroinvertebrates (Padial et al., 2014). It is recommended that *Spatial* factors should be included when considering factors influencing biodiversity patterns (Grenouillet et al., 2008). Geo‐climatic (*Geo*) change puts tremendous survival pressure on terrestrial and aquatic organisms and disrupts biodiversity (Buisson & Grenouillet, 2009; Haeder & Barnes, 2019), affecting the structure and function of the ecosystem (Comte et al., 2013). Studying the role of *Local*, *Geo*, and *Spatial* factors on fish communities can help to understand how species are affected by environmental filtering and dispersal limitations at spatial scales (Pelaez & Pavanelli, 2019). However, few studies have simultaneously examined the interaction of *Local*, *Geo*, and *Spatial* factors. This paper studies the relative and combined contribution of abiotic factors to various facets of fish *β*‐diversity and their components to explore the main abiotic factors driving local fish and the potential impact on the operational mechanisms of fish *β*‐diversity (Gianuca et al., 2017).

Organisms tend to exhibit the same traits in the same environment, and species replacement in taxonomy is not necessarily associated with trait turnover (Heino & Tolonen, 2017; Parreira de Castro et al., 2018). Hence, we hypothesize that (i) in the total *β*‐diversity facet, functional *β*‐diversity supposedly be relatively low compared with phylogenetic and taxonomic *β*‐diversity, especially in the turnover facet (H1); (ii) we also hypothesized a high correlation between taxonomic and phylogenetic *β*‐diversity compared with the other two combinations (H2; Li, Olden, et al., 2020); (iii) several researchers have demonstrated that *Spatial* factors are more important in populations with low dispersal ability (Pelaez & Pavanelli, 2019; Vardakas et al., 2020). Fish in the Hun‐Tai River basin are affected by low river connectivity, migration, and spatial longitudinal gradients, resulting in a weak dispersal capacity (Liu & Wang, 2018; Padial et al., 2014; Wu et al., 2021). We presume that *Spatial* factors should be more significant in contributing to fish *β*‐diversity than *Local* and *Geo* factors (H3).

## 2 METHODS

### 2.1 Study area description

The Hun‐Tai River is a sub‐basin of the Liao River, consisting of the Hun River and the Taizi River, and is located in Liaoning Province in northeastern China. (40°40′–42°10′N, 122°5′–125°17′E), with a basin area of 27,300 km^2^, a temperate monsoon climate, the average annual temperature is 9°C, and an average annual rainfall is 686.4 mm (Wang et al., 2018; Wu et al., 2021; Zhang et al., 2017).

This basin is ideal for us to explore facets of fish *β*‐diversity and their driving mechanisms (Wu et al., 2021; Zhang et al., 2017). First, the basin has a large spatial scale and significant changes in elevation gradients and climatic environments (Panja et al., 2021). It is helpful for studying the taxonomic composition, functional traits, and community phylogenetics of freshwater fishes in response to the longitudinal gradient of rivers, geo‐climatic, and local environments (Buisson & Grenouillet, 2009; Wang et al., 2010). Second, the research field can better compare the impact of human activities on biodiversity (Li, Tan, et al., 2020). The middle and upper reaches of the Hun‐Tai River have high forest coverage, with higher levels of developed industries and agriculture in the lower reaches (Qian et al., 2017), and the upper and lower reaches are affected by different degrees of human disturbance (Li, Tan, et al., 2020; Zhang et al., 2017). Third, water quality in the basin has improved as a result of the measures taken by the environmental department to deal with water pollution in the key basins (Kang et al., 2020). Nonetheless, nonpoint source pollution from agricultural lands and urban outputs will be a crucial factor influencing water quality in the basin (Nobre et al., 2020). Most researchers have concerned themselves with the relationship between riverine organisms (fish, macroinvertebrates, and benthic algae) and environmental factors in the basin (Wu et al., 2021), as well as the ecosystem integrity assessment (Wang et al., 2018). However, little research has focused on the relative contribution of each component of fish *β*‐diversity (Qian et al., 2021), and the main drivers of these components (Wu et al., 2021). Our study analyzed the main driving mechanisms affecting the taxonomic, functional, and phylogenetic *β*‐diversity of fish communities (turnover and nestedness), and explored the potential impact of the interaction of three abiotic factors (*Local*, *Geo*, and *Spatial* factors) on fish *β*‐diversity, which differs from other studies (Benone et al., 2020; Qian et al., 2021).

### 2.2 Field sampling and processing

**FIGURE 1:**
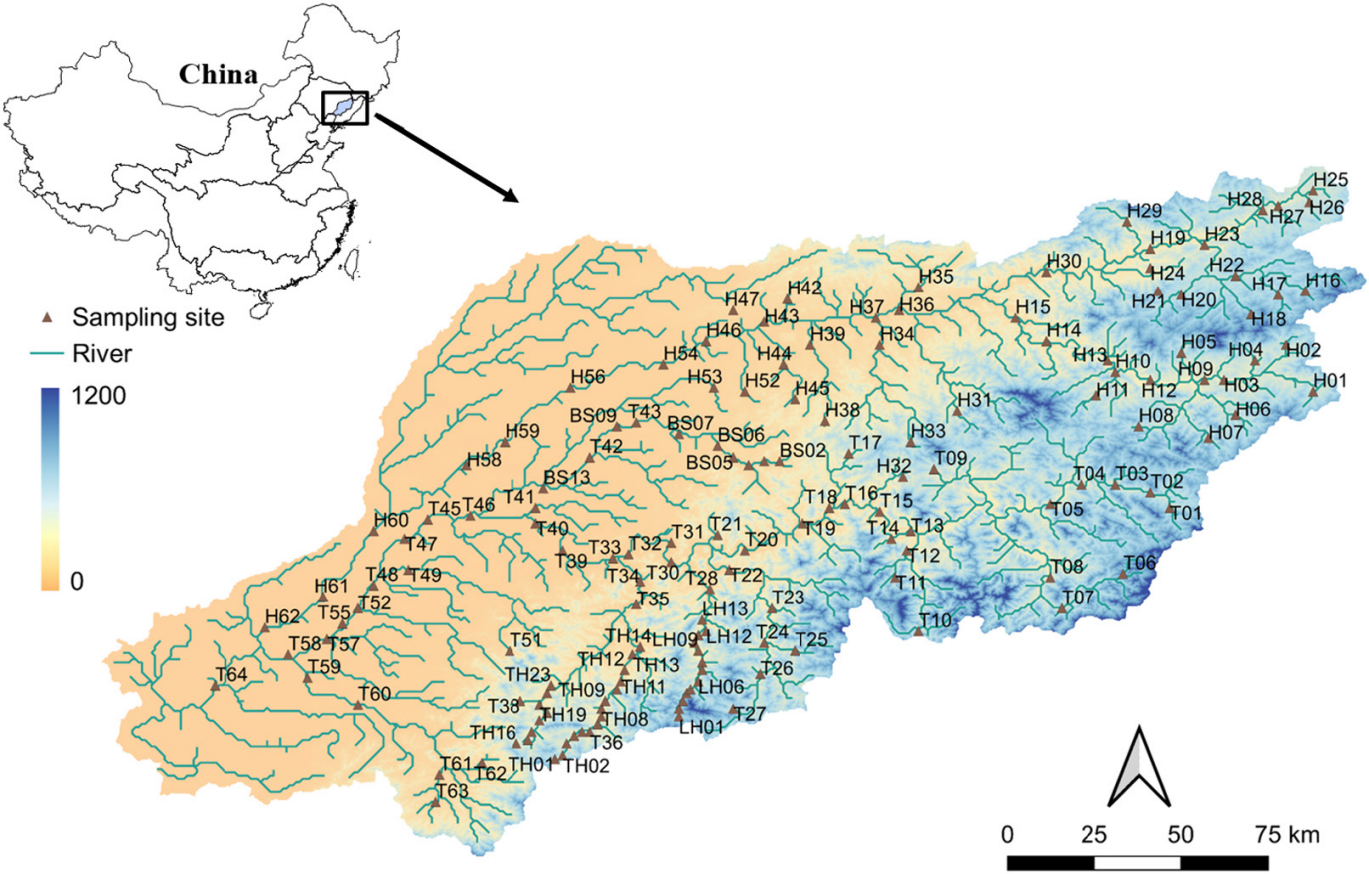
The distribution of 171 sampling points in the Hun‐Tai basin in Northeast China. The letters H, TH, and BS in the figure represent different basins. BS, Benxi river; H, Hun River; LH, Liaohe river; T, Taizi river; TH, Tanghe river.

A total of 171 sampling points were selected in the Hun‐Tai River, and physical, chemical, and hydrological factors were measured at all sampling sites (Figure 1). At each sampling point, we recorded geographic coordinates (GPS receiver) and utilized the YSI Multiparameter (Pro Plus) instrument to measure water pH, water temperature (WT), dissolved oxygen (DO), conductivity (Cond), and total dissolved solids (TDS). River width (width), in situ water depth (depth), and flow velocity (velocity; global water flow probe FP201) were also measured. In addition, the habitat quality assessment index (QHEI) reflects the quality of the measured habitat by characterizing the physical habitat of the stream (Stewart & Scott, 1995). QHEI indices are divided into 10 categories: substrate composition, range of integrated water depths and flow rates, in‐stream habitat complexity, channel sinuosity, bank stability, biodiversity of riparian plants, water quantity, environmental pressure from human activities, visual inspection of water cleanliness, and land use types (An et al., 2002; Santucci et al., 2005). Each classification was visually evaluated on a scale of 0–20, indicating the quality of the habitat from low to high. The final sum of these 10 categories is the QHEI score. Surface water samples from the collected streams were acid‐fixed and analyzed in the laboratory for water chemistry such as soluble reactive phosphorus (PO_4_‐P), nitrite nitrogen (NO_2_‐N), chemical oxygen demand (COD), total nitrogen (TN), ammonia nitrogen (NH_4_‐N), suspended solids (SS), and total phosphorus (TP). TN/TP (NPR) calculations are the ratio of TN to TP. Zhou et al. (2020) and Qu et al. (2019) have previously described in detail the measurement of water chemistry.

We collected fish from 171 sample sites and fished with a self‐regulating backpack‐style electric fishing device with two rechargeable lithium‐ion batteries (29 V) and a control system. The sampling time was limited to 30 min, and the interval length of each sampling point is approximately 500 m. Two sheets of nylon monofilament‐barbed wire (mesh size: 10 mm) were set up to block the upper and lower boundaries of the river section prior to sampling in the field. Fish were immediately identified, counted, and weighed according to local standard references (Qu et al., 2019; Xie, 2007), whereas unidentified fish samples were brought back to the laboratory for identification.

### 2.3 Geo‐climatic variables

Geo‐climatic variables consist primarily of topography, climate, and land use type. Topographic data and land use data are based on previous studies from www.earthenv.org (Amatulli et al., 2018; Domisch et al., 2015; see Appendix S1 for details). We calculated the percentage of each land use type occupied by each sampling point in the upstream watershed. Land use data were from the consensus landcover data set (Bontemps et al., 2012; Tuanmu & Jetz, 2014). A total of 12 categories of land cover were found in the original data set. This study combines deciduous broadleaf trees, mixed/other trees, evergreen/deciduous conifers, and evergreen broadleaf trees into one category “forest”. Furthermore, no herbaceous and shrub vegetation that is frequently flooded is found in this watershed. Hence, a total of eight land use types were analyzed (i.e., herbaceous vegetation, shrubs, forest, urban, agriculture, snow/ice, open water, and barren lands).

Moreover, the terrain information of each sampling point was extracted (e.g., elevation, gradient, slope). The slope represents the steepness of the river along the longitudinal gradient, whereas the aspect ratio captures the east–west and north–south direction of each location (Amatulli et al., 2018). Nineteen bioclimatic variables (Bio1 to Bio19) were obtained from the WorldClim 2 database (Fick & Hijmans, 2017). The data obtained for each sampling site include annual precipitation, average annual temperature, minimum temperature (the coldest month), and maximum temperature (the hottest month). The database was averaged for 1970–2000 at a spatial resolution of 1 km (Appendix S1).

### 2.4 Species traits

We constructed fish species traits for the Hun‐Tai River based on published literature, recorded 42 fish species in the study area, and selected 14 functional traits such as fish body morphology (teeth, mouth, caudal, or beard) and life history (diet, habit, spawning, or adaptation temperature) to quantify functional space (Poff & Allan, 1995; Table 1). These traits were chosen for the following three reasons: (a) preserve as much information as possible about the fish species available for a more complete description of the functional space; (b) these traits reflect fish physical traits, food access, habitat preference, spawning, and trophic levels; (c) all these biological traits are related to the conditions under which they live in freshwater systems. These traits help us to study functional *β*‐diversity in fish (Sternberg & Kennard, 2013).

**TABLE 1:** Fish traits, their categories, codes, and descriptions used in this study

Traits	Categories	Codes	Descriptions
1. Body morphology (Olden et al., 2004)	Teeth type	TTY	Connected to species' feeding and diet composition (Karachle & Stergiou, 2011)
	Mouth type	MTY	
	Caudal type	CTY	Position and locomotion of fish in currents (e.g., swimming, steering, maneuvering; Gosline, 1997)
	Beard type	BTY	
	Maximum body length	MBL	Related to individual biomass, dispersal, abundance, metabolic rate, and food web Location (Costello et al., 2015; Sternberg & Kennard, 2013)
	Length–weight ratio	LWR	
	Body Type	BTY	Habitat, activity, and location in the river (Henseler et al., 2019)
2. Life history Included (Olden et al., 2004)	Phylogenetic diversity 50	PD50	Represents how species are distributed within a phylogenetic tree (Pavoine et al., 2009; https://www.fishbase.de/)
	Protection Function	PFU	Egg protective behavior of fish after spawning (La Mesa et al., 2021)
	Spawning	SPA	Reproduction of the offspring of the species, key vulnerable events (Miller et al., 2015)
	Habit Type	HTY	Living environment type of fish habitat (Leal et al., 2021)
	Adaptation Temperature	ATE	Affects fish metabolism and growth rates as well as the social behavior of fish in calm water (Bartolini et al., 2015)
	Trophic Level	TLE	Types and frequency of nutritional objects in fish diet (Costello et al., 2015)
	Diet	DIE	Feeding type, food acquisition, ecological niche occupation (Costello et al., 2015)

### 2.5 Data analysis

We used R (version 4.0.2, R Core Team, 2020) for all data analysis. The calculation of *Spatial* factor is based on the principal coordinates of neighbor matrices (PCNM; it is the *pcnm* function in R package *vegan*; Oksanen et al., 2019) and distance‐based Moran eigenvector map (MEMs; it is the *dbmem* function in R package *adespatial*; Dray et al., 2021) and describes the spatial structure of the data set. We used MEMs analysis because we found a significant correlation between PCNMs and MEMs (Mantel test: *r* = .424, *p* = .001).

We divide the obtained data sets into abiotic and biotic data sets. The abiotic data set includes (i) *Local* factors, including 18 physicochemical variables measured in the field and laboratory (Appendix S1); (ii) *Geo* factors, including three topographic variables (elevation, slope, and aspect), eight land use types, and 19 bioclimatic variables (Bio1‐19); and (iii) *Spatial* factors, including 45 MEMs. The biotic data set includes (i) taxonomic *β*‐diversity matrices, using the *beta. pair* function in the R package *betapart* (Baselga & Orme, 2012); (ii) functional *β*‐diversity matrices, in which total *β*‐diversity is divided into turnover and nestedness components using the function *functional.beta.pair* in the R package *betapart*; (iii) phylogenetic *β*‐diversity matrices, using the *phylo*.*beta.pair* function in the R package *betapart*. These *β*‐diversity decompositions enabled us to examine the relative contributions of turnover and nestedness components to functional, taxonomic, and phylogenetic *β*‐diversity (Pool et al., 2014).

We ran Mantel tests (with 999 permutations) to examine the relationship between functional, taxonomic, and phylogenetic *β*‐diversity components. The Mantel statistic *r* (ranging from −1 to 1) stands for a correlation between two dissimilarity or distance matrices, with a higher Mantel *r* indicating a stronger correlation. Mantel tests were conducted using the function *mantel* in the R package *vegan*. To examine if variation in each facet of *β*‐diversity and its components were related to *Local*, *Geo*, or *Spatial* factors, we performed distance‐based redundancy analysis (db‐RDA; Legendre & Anderson, 1999) on each facet of *β*‐diversity and its components.

Before using each data set (i.e., three abiotic factors), we deleted the variables with significant multicollinearity (variance inflation factor, VIF) ≥ 3 based on the *vifstep* function in the R package *usdm* (Naimi et al., 2014). Those variables that did not show strong collinearity were included in db‐RDA (using the function *capscale* in R package *vegan*). We tested the marginal significance of each variable in the model, the overall significance of the ordination solutions, and the amount of explained variation (*R* ^2^). In all db‐RDA analyses, the *sqrt.dist* correction for negative eigenvalues was employed as in previous studies (Perez Rocha et al., 2018; Wu et al., 2021). We applied variation partitioning analysis (VPA; Borcard et al., 1992) to quantify the relative contributions of three abiotic factors to each facet of *β*‐diversity and its components which have been widely used in ecology to determine ecological processes (Lopez‐Delgado et al., 2020). Forward selection (using the *forward.sel* function in R package *adespatial*) was conducted to obtain a final set of *Local*, *Geo*, and *Spatial* factors with two stopping criteria: the adjusted coefficient of determination (adjusted *R* ^2^) and significance level (Blanchet et al., 2008). The significance of the pure fractions was tested using the *anova* function in the R package *vegan* at a significance level of *α* = 0.05. VPA was carried out with the *varpart* function in the R package *vegan*.

## 3 RESULTS

### 3.1 Three facets of *β*‐diversity decomposition

**FIGURE 2:**
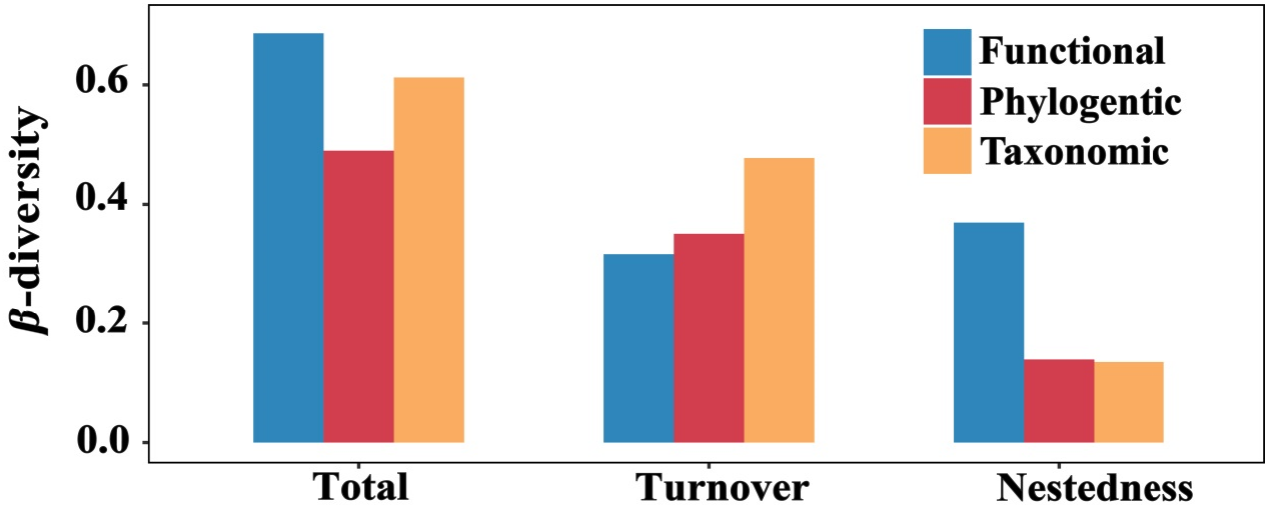
Three facets (taxonomic, functional, and phylogenic) of the *β*‐diversity component (i.e., total, turnover, and nestedness) of fish communities in the Hun‐Tai River basin.

A total of 42 species of fish were observed in this study (see Appendix S2 for a list of species), and the average species abundance per site was 5.96. From our *β*‐diversity decomposition calculation, we found that total functional *β*‐diversity (mean ± SD: 0.686 ± 0.245) was driven by nestedness (mean ± SD: 0.370 ± 0.315) and complemented by turnover (mean ± SD: 0.317 ± 0.321). Total phylogenetic *β*‐diversity (mean ± SD: 0.490 ± 0.151) was mainly contributed by turnover (mean ± SD: 0.351 ± 0.192) and less by nestedness (mean ± SD: 0.139 ± 0.119). Total taxonomic *β*‐diversity (mean ± SD: 0.612 ± 0.195) was driven by turnover (mean ± SD: 0.478 ± 0.257), with a less contribution of nestedness (mean ± SD: 0.135 ± 0.131; Figure 2). To sum up, total functional *β*‐diversity was higher than total taxonomic and phylogenetic *β*‐diversity, which conflicted with our expectations (H1).

**FIGURE 3:**
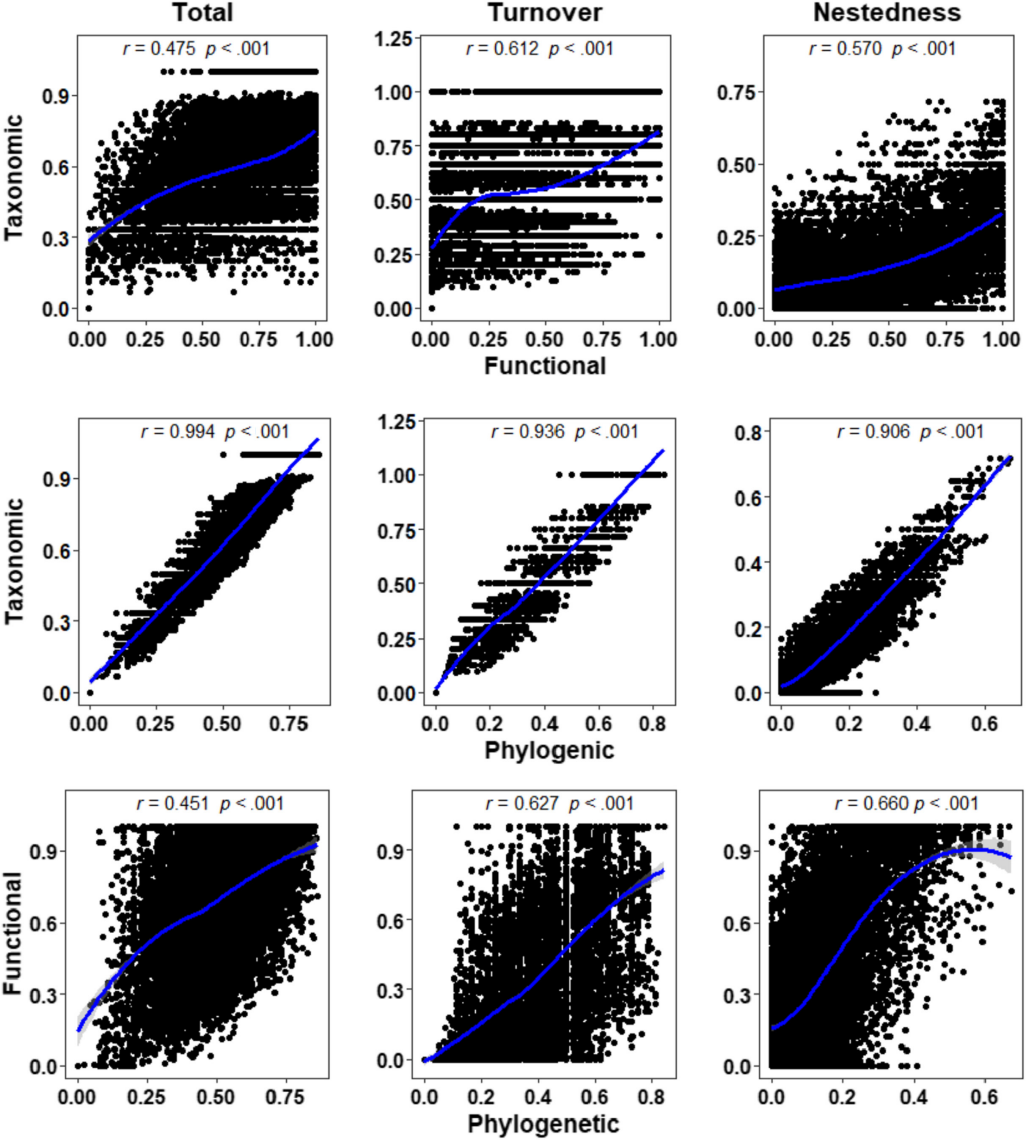
Relationships between taxonomic, functional, and phylogenetic *β*‐diversity components of the fish community (i.e., total *β*‐diversity, turnover, and nestedness), in which *r* is the Mantel coefficients and the significance level were tested using the Mantel test (*p* < .001); the solid blue lines were the LOESS smoothing and shaded gray areas were 95% confidence interval of the fit.

In the results of Mantel tests for *β*‐diversity facets and its components, significant correlations (*p* < .001) between functional, taxonomic and phylogenetic *β*‐diversity were observed (Figure 3). Among them, based on the total *β*‐diversity, the correlation between taxonomic and phylogenetic facets was the highest (*r* = .994); the highest correlation between functional and taxonomic *β*‐diversity was for turnover (*r* = .612); the highest correlation between the phylogenetic and functional *β*‐diversity was nestedness (*r* = .660). To summarize, the correlation was significant in all taxonomic and phylogenetic *β*‐diversity, which confirmed our second hypothesis (H2).

### 3.2 Main drivers of taxonomic, functional and phylogenetic *β*‐diversity components

The components of *β*‐diversity were all different in the selected variables of the db‐RDA model (Table 2; see Appendix S3–S5 for details). We used a forward selection procedure and a multicollinearity test to select abiotic factors in terms of taxonomic *β*‐diversity components: 9 *Local*, 8 *Geo*, and 12 *Spatial* factors for total; 8 *Local*, 9 *Geo*, and 12 *Spatial* factors for turnover; 2 *Local*, 5 *Geo*, and 6 *Spatial* factors for nestedness; fewer variables were selected for functional *β*‐diversity, especially *Local* and *Geo* factors; 3 *Local*, 3 *Geo*, and 9 *Spatial* factors for total; 5 *Local*, 7 *Geo*, and 12 *Spatial* factors for turnover; 4 *Local*, 3 *Geo*, and 10 *Spatial* factors for nestedness; based on phylogenetic *β*‐diversity, the following were selected: 9 *Local*, 7 *Geo*, and 12 *Spatial* factors for total; 8 *Local*, 7 *Geo*, and 11 *Spatial* factors for turnover; 2 *Local*, 4 *Geo*, and 10 *Spatial* factors for nestedness. The selected *Local* variables represent mainly hydrological conditions (e.g., river width, depth), habitat quality (e.g., QHEI), and water chemistry (e.g., COD, NH_4_‐N). The selection of Geo variables includes climatic conditions (e.g., Bio14: isotherms; precipitation in the driest months), land use type (e.g., urban, herbaceous), and topographic parameters (e.g., slope). For the *Spatial* factor, the selection was mainly spread over 25 PCNMs, indicating that it was influenced by the wide spatial scale variation.

**TABLE 2:** Local environmental (*Local*), geo‐climatic (*Geo*), and spatial (*Spatial*) factors, based on the results of positive selection of *β*‐diversity facets and components.

*β*‐Diversity facets	Variables	Total	Turnover	Nestedness
Taxonomic	Local	*n* = 9***	*n* = 8***	*n* = 2*
	Geo	*n* = 8***	*n* = 9***	*n* = 5***
	Spatial	*n* = 12***	*n* = 12***	*n* = 6**
Functional	Local	*n* = 3***	*n* = 5***	*n* = 4*
	Geo	*n* = 3***	*n* = 7***	*n* = 3**
	Spatial	*n* = 9***	*n* = 12***	*n* = 10**
Phylogenetic	Local	*n* = 9***	*n* = 8***	*n* = 2
	Geo	*n* = 7***	*n* = 7***	*n* = 4***
	Spatial	*n* = 12***	*n* = 11***	*n* = 10**

*Note*: Number (*n*) is the significant factor by forward selection and significance is indicated as *p < .05, **p < .01, ***p < .001 (see Appendix S3–S5 for details).

**FIGURE 4:**
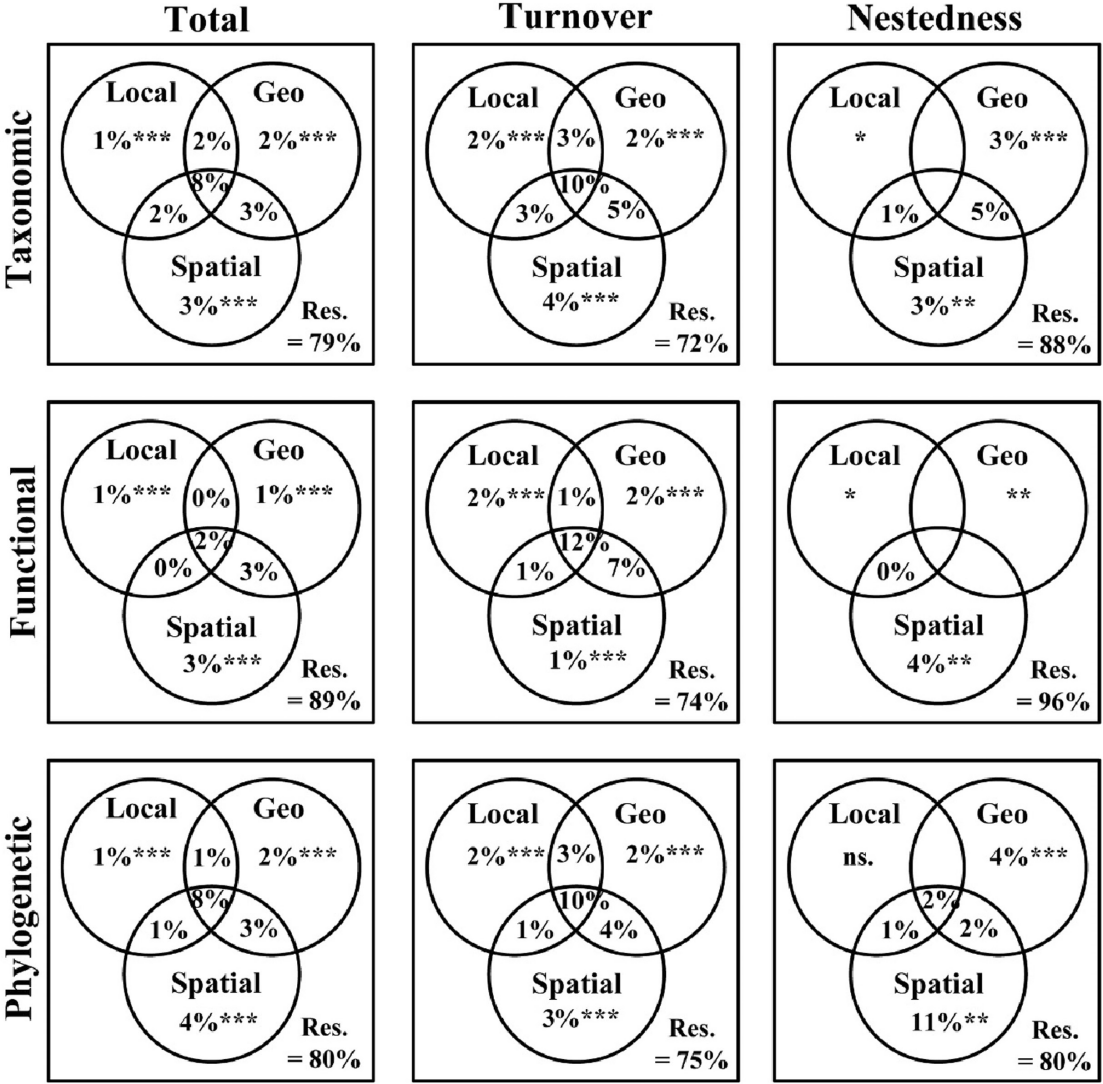
Relative contributions of local environmental (*Local*), geo‐climatic (*Geo*), and spatial (*Spatial*) factors to the taxonomic, functional, and phylogenetic *β*‐diversity components (total, turnover, and nestedness). The adjusted *R* ^2^ was used (values < 0 are not shown); ns, represents not significant; res expresses unexplained variation (total variation = 100). ***p < .001, **p < .01, *p < .05.

Analysis of VPA shows that all three individual factors are statistically significant (*p* < .001): *Local*, *Geo*, and *Spatial* factors (Figure 4). (i) The results show that the pure contribution of *Geo* factors (1%–4%) is slightly more important than the contribution of *Local* factors (1%–2%) but less important than the *Spatial* factors (1% ~ 11%); (ii) based on taxonomic, functional, and phylogenetic *β*‐diversity and their components (total, turnover, and nestedness), the combined impacts of the *Local*, *Geo*, and *Spatial* factors were 8%, 10%, 2%, 12%, 8%, 10%, and 2%, respectively. This suggests an important interaction between these three abiotic factors; (iii) based on taxonomic *β*‐diversity, the model explains 21% total, 28% turnover, and 12% nestedness. Based on functional *β*‐diversity, the model explains 11% total, 26% turnover, and 4% nestedness. Based on the phylogenetic *β*‐diversity model explains 20% of total, 25% turnover, and 20% nestedness. In summary, as expected by H3, *Spatial* factors contribute more to *β*‐diversity than *Geo* and *Local* factors, and the combined effect of these three factors drives the highest turnover.

## 4 DISCUSSION

### 4.1 Contribution of turnover and nestedness

Studying the contribution of *β*‐diversity from different facets is crucial to analyzing the causality of potential biodiversity processes (Gianuca et al., 2017; Taylor, 2010; Villeger et al., 2013). The results showed that functional *β*‐diversity was higher than taxonomic and phylogenetic *β*‐diversity (0.686 vs. 0.612 vs. 0.490), this was not our expectation but is consistent with recent findings on fish (Araujo et al., 2019; Jia et al., 2020). High functional *β*‐diversity (Figure 2) may have the following two reasons: (i) low abundance of Hun‐Tai river fish species compared with algae and macrobenthos (Qu et al., 2019; Wu et al., 2021), species with substantial overlap between combinations (e.g., certain common species occur multiple times in fish assemblages); (ii) fish of the same genus may have different functional traits. For example, two fishes of the same genus (*Etheostoma flabellare* and *E. olmstedi*) have interspecific differences in performance (Carlisle et al., 2008). Compared with taxonomic and phylogenetic facets, higher functional *β*‐diversity shows that different species often play distinct functions in spatial environment combination (Araujo et al., 2019).

High nestedness of functional *β*‐diversity suggests that differences in species functional richness (Baselga, 2010) may be due to several reasons: (i) local extinction can lead to the creation of nestedness combinations (Wang et al., 2010; Wu et al., 2022). For instance, some rare fish species with special functions have high requirements for habitat water quality and become extinct due to habitat destruction; (ii) high‐functioning communities contain the functional traits of low‐functioning communities. For example, some functional communities are species‐rich, and less functional communities are subsets of them (Bender et al., 2017); (iii) combinations of some functional traits may be represented by several species (Matthews et al., 2015). Lujan and Conway (2015) found that rheophilic fishes often have streamlined bodies or morphological traits that facilitate survival at high altitudes and rushing rivers. Overall, the low functional turnover rate and high functional nestedness indicated that the trait of fish in this study area was more homogeneous (Wu et al., 2021). Studying the operational mechanisms of functional *β*‐diversity helps to clarify community aggregation processes on environmental gradients or spatial scales (Devictor et al., 2010; Lamothe et al., 2018; Villeger et al., 2013).

In addition, we found that turnover dominates fish taxonomic and phylogenetic *β*‐diversity, suggesting the predominance of species replacement over species loss (Araujo et al., 2019). Similar patterns have been observed in previous studies on fish (Liu & Wang, 2018; Lopez‐Delgado et al., 2020). Metacommunity theory reveals that community structure is determined not only by local abiotic environmental conditions but also by biotic interactions and dispersal‐related effects (Heino, 2013). First, fish in the Hun‐Tai River are constrained by mountain barriers, fragmented river networks, and elevation and have strong dispersion restrictions (e.g., only fish with high dispersal ability can reach more habitats). This means that there is dispersed isolation between species (e.g., the proportion of shared species between two communities with similar numbers of species is relatively low; Villeger et al., 2013). Second, the formation of species in a wide space is more likely to lead to the emergence of turnover patterns (e.g., adult fish can make moderate to large‐scale migrations in a wide basin; Villeger et al., 2013). Dobrovolski et al. (2012) found that differences between assemblages are driven primarily by variations in species composition between highly isolated sites. The advantage over taxonomic turnover for taxonomic nestedness (0.478 vs. 0.135) illustrates that heterogeneity in fish assemblages is primarily due to species substitution rather than differences in richness (Pelaez & Pavanelli, 2019).

### 4.2 Relationship between functional, phylogenetic, and taxonomic *β*‐diversity

Studying the taxonomic, functional, and phylogenetic facets relationship of species at the river basin and local scales helps to understand the ecological information linkages among the various components of *β*‐diversity and provides a complementary approach to studying biodiversity patterns (Baselga, 2010; Huang et al., 2020; Steudel et al., 2016). The results showed significant correlations between functional and taxonomic *β*‐diversity, which may indicate that as species richness increases, the functional composition of the community will be more diverse. This is consistent with recent findings on *β*‐diversity in fish (Colin et al., 2022). Moreover, taxonomic and functional *β*‐diversity showed significant correlations in turnover may be due to species turnover reducing the redundancy of species functional assemblages and increasing the stochasticity of functional trait assemblages (Lamothe et al., 2018). It means that the ecological connection between the spatial distribution and function of species will be closed when turnover is the main driving mode.

Another interesting finding was that the correlation between taxonomic and phylogenetic *β*‐diversity was extremely strong (Mantel *r* range .906 ~ .994), and some researchers have recently confirmed the same results (Qian et al., 2021; Tsianou et al., 2021), supporting our second hypothesis (H2). Branco et al. (2020) pointed out that taxonomic and phylogenetic *β*‐diversity are scale correlated. In particular, taxonomic and phylogenetic facets showed an extremely high correlation in total *β*‐diversity (0.994; Qian et al., 2021). This is likely due to the homogenization of human‐disturbed river habitats, resulting in a high degree of taxonomic and phylogenetic facets convergence of fish (Taylor, 2010; van der Plas et al., 2016; Villeger et al., 2015). Recent studies on phylogenetic and taxonomic (Olden et al., 2018) *β*‐diversity have yielded similar results (Thuiller et al., 2011). They have indicated that anthropogenic disturbance has become one of the key factors affecting community composition. Given the important impact of biodiversity on ecological and evolutionary processes (Olden et al., 2004) and ecosystem versatility (van der Plas et al., 2016), future studies shall focus on the response of various facets of biodiversity to external environmental disturbances.

Furthermore, functional and phylogenetic *β*‐diversity showed a significant correlation in the nestedness component. This shows that the functional and phylogenetic facets of fish communities are facing different degrees of diversity loss. Correlation analysis of functional and phylogenetic *β*‐diversity among species can be a better understanding of ecological similarities and differences among proximate species (Huang et al., 2020). From the above findings, the functional *β*‐diversity of fish is more vulnerable to loss (Lin et al., 2021).

### 4.3 Contribution of three abiotic factors to *β*‐diversity components

*Local*, *Geo*, and *Spatial* factors drive patterns of variation in species and trait composition among habitats (Buisson & Grenouillet, 2009; Goncalves et al., 2020; Lopez‐Delgado et al., 2020; Zhou et al., 2020). At present, little is known about how these abiotic factors respond to simultaneous changes in aquatic communities. While in the past, it was common to analyze the effects of individual factors on organisms, it is becoming increasingly apparent that the superposition, synergism, or antagonism of all these factors in the environment must be considered (Haeder & Barnes, 2019). In the system of metacommunities, the role of interactions between spatial and environmental processes in shaping the composition of local communities is clear (Padial et al., 2014; Pelaez & Pavanelli, 2019). If environmental variables mainly predict community composition, then mechanisms related to ecological niches are considered the main drivers of assembled communities, with species being classified in different habitats (Heino & Mykra, 2008). Another view emphasizes that the differences in the structure of local communities are mainly due to random processes, including diffusion limitation and ecological drift. Our results showed that the main driver was the *Spatial* factors, and the other two factors contributed weakly (Soininen et al., 2018). From previous investigations, we found that *Local* factors in flat watersheds or tropical oceans have a strong effect on each fish component (Leprieur et al., 2012; Lopez‐Delgado et al., 2020), but in watersheds with high elevation gradients and spatial patterns with more complex, Spatial are often the main drivers of dominant river biodiversity (e.g., fish, benthic algae, macroinvertebrates; Perez Rocha et al., 2018; Vardakas et al., 2020; Zhou et al., 2020). The large longitudinal gradient and high elevation of the Hun‐Tai River shape the complex spatial pattern (Zhou et al., 2020). The taxonomic composition, phenotypic traits, and community structure of freshwater fishes worldwide are strongly influenced by the longitudinal river gradient (Lujan et al., 2013). Therefore, elevation may affect the species composition of fish more directly through dispersal limitation (Shurin et al., 2009). At the same time, the combination of different temperature gradients in the basin also indirectly affects the reproduction of fish (Boll et al., 2016). Moreover, the comparative importance of deterministic (e.g., species classification) and stochastic processes (e.g., dispersal) will depend on the dispersal ability of the population of organisms under study (Padial et al., 2014). As specialized aquatic organisms, fish must use river networks for locomotion and may show higher spatial process signals in diversity patterns than less physically constrained organisms (Padial et al., 2014; Vitorino et al., 2016). As described in this research, species are susceptible to dispersal limitations (Leibold et al., 2004). In freshwater ecosystems, dispersal capacity is usually inversely proportional to body size (Costello et al., 2015; Karachle & Stergiou, 2011; Shurin et al., 2009). Therefore, fish dispersal capacity may be relatively low. Our results show that *Spatial* factors are the main drivers of local communities for fish biota and that fish community composition is more stochastic. This conclusion was also demonstrated in many different species, habitat types, and geographic regions (Pelaez & Pavanelli, 2019; Perez Rocha et al., 2018; Wu et al., 2021), which supports our third hypothesis (H3). Boschilia et al. (2016) and Soininen et al. (2018) suggest that the high species turnover among communities, which may reflect the selection of species by spatial filters, is consistent with our conclusion and also reflects the impact of *Spatial* factors on species *β*‐diversity mode.

We also found that the interaction and contributions of *Geo* and *Local* factors are the same in three facets of *β*‐diversity (total). Climate change exposes aquatic organisms to high levels of human‐induced stress (Haeder & Barnes, 2019). For example, seasonal patterns of precipitation influence the chemical fluxes of aquatic ecosystems, water flow, solar radiation, and lateral floodplain recharge, thus affecting aquatic community structure (Haeder & Barnes, 2019). Moreover, the survival of fish depends largely on the seasonal precipitation of river flow and land landscape (Perkin et al., 2013). As the global warming trend has been increasing and ocean temperatures are rising (Reguero et al., 2019), freshwater has also been affected, with a decrease in the amount and area of suitable habitats for most cold‐water fishes, and local extinctions and distribution contractions are also expected in the future (Comte et al., 2013). Thus, *Geo* factors will be an essential predictor of future aquatic research.

In this study, the contribution of *Local* factors was weak compared to the other two factors. We also found that the single contribution of *Geo* factors is the same in three facets of *β*‐diversity (turnover), which is not reflected in the nestedness component. The upper reaches of the Hun‐Tai River are affected by large elevation changes and mountain barriers, with low species richness, and the lower reaches are affected by fishing pressure, farmland pollution, and hydropower construction. These human disturbances result in river fragmentation, weak flood pulsation, and low connectivity (Castello & Macedo, 2016). These environmental changes and human disturbances are disrupting the biodiversity, structure, and function of ecosystems, undermining their ability to provide food and vital ecological services to humans (Comte et al., 2013; Jia et al., 2020; Steudel et al., 2016), thereby exposing fish fauna to a reproductive and survival crisis. Our results indicate that *Spatial* factors are the main mechanism affecting fish fractions and *β*‐diversity. Dispersal ability dictates the primary role of environmental, spatial, and climatic drivers on community structure (Padial et al., 2014). Ecological niche filtering and spatial connectivity explain the higher functional nestedness component (Pelaez & Pavanelli, 2019). High turnover rates in taxonomic, phylogenetic, and functional facets are explained by the shared ratio of the three abiotic factors (turnover ≥ 10%). This indicates that the comprehensive role of *Local*, *Geo*, and *Spatial* factors has a strong influence on species turnover (Vitorino et al., 2016).

## 5 MANAGEMENT INSIGHTS AND CONCLUSIONS

We investigated the correlation of taxonomic, functional, and phylogenetic *β*‐diversity in freshwater fish communities and the main driving mechanisms and compared the relative contribution of three abiotic factors in these facets, with three main findings: (i) turnover rate is the main driving mechanism for the *β*‐diversity of fish in the Hun‐Tai river, especially in fish taxonomic and phylogenetic facets. The high turnover rate in taxonomic *β*‐diversity indicates the need to protect the whole basin (Wu et al., 2021). High functional nestedness may mean that higher functional diversity can be maintained by preserving richer loci (Lin et al., 2021; Socolar et al., 2016). By measuring biodiversity, it is possible to understand its determinants and prioritize conservation (Devictor et al., 2010; Li, Olden, et al., 2020; McKnight et al., 2007); (ii) there is a strong correlation between fish phylogenetic and taxonomic *β*‐diversity. Compared with more traditional taxonomic methods, such studies enhance the explanatory power of *Local*, *Spatial*, and *Geo* related variables (Buisson & Grenouillet, 2009; Heino & Tolonen, 2017; Vardakas et al., 2020). This supports the view that research on functional traits and phylogenetic *β*‐diversity can help to better understand ecological similarities and differences among closely related species (Cassia‐Silva et al., 2019; Perez Rocha et al., 2018; Qian et al., 2020); (iii) by analyzing the relative contribution of three abiotic factors to *β*‐diversity, we found that *Spatial* factors promote the development of nestedness patterns, and environmental variability may be more important to the functional differences between different locations (Hill et al., 2019; Pease et al., 2015). Connectivity may be a factor influencing differences in taxonomic and functional richness (Pelaez & Pavanelli, 2019). In addition, considering the complexity of river networks and their physical constraints from a metacommunity perspective can improve understanding of the role of local and regional processes in driving patterns of freshwater system diversity (Appolloni et al., 2017; Heino & Tolonen, 2017; Padial et al., 2014; Vardakas et al., 2020).

In conclusion, our findings emphasize that the study of *β*‐diversity facets and their components is crucial for the conservation of local freshwater fishes. In particular, the high correlation between phylogenetic and taxonomic *β*‐diversity provides similar ecological information for ecosystem conservation and management (Socolar et al., 2016). In the future, it will be better to study temporal *β*‐diversity (e.g., appropriate time scales).

## AUTHOR CONTRIBUTIONS

**Naicheng Wu:** Conceptualization (equal); formal analysis (lead); methodology (equal); writing – original draft (supporting); writing – review and editing (equal). **Yuanyuan Lv:** Formal analysis (equal); methodology (equal); writing – original draft (lead); writing – review and editing (equal). **Min Zhang:** Conceptualization (equal); data curation (supporting); investigation (equal); writing – original draft (supporting); writing – review and editing (equal). **Yaochun Wang:** Formal analysis (supporting); writing – original draft (supporting); writing – review and editing (equal). **Wenqi Peng:** Conceptualization (supporting); funding acquisition (supporting); investigation (equal); resources (equal); writing – original draft (supporting); writing – review and editing (equal). **Xiaodong Qu:** Conceptualization (equal); formal analysis (equal); funding acquisition (lead); investigation (equal); methodology (equal); resources (equal); writing – original draft (equal); writing – review and editing (equal).

## FUNDING INFORMATION

This study was supported financially by the National Natural Science Foundation of China (nos. 52279068 and 32071588), the National Key R&D Program of China (no. 2021YFC3200102), and Starting Grants of Ningbo University (nos. 421999292, 422110123, and 422205193).

## CONFLICT OF INTEREST

The authors declare that they have no conflict of interest to disclose.

## Supporting information


Appendix S1:

## Data Availability

All data are deposited in the Dryad repository upon acceptance https://doi.org/10.5061/dryad.wstqjq2pj.
