# Supplementary material for: Understanding the relative roles of local environmental, geo‐climatic and spatial factors for taxonomic, functional and phylogenetic β‐diversity of stream fishes in a large basin, Northeast China
Source: Ecol Evol. 2022 Dec 12;12(12):e9567. doi: 10.1002/ece3.9567 (PMC9745106; doi:10.1002/ece3.9567)
Supplement: Supplementary file 1 — Appendix S1: [file ECE3-12-e9567-s001.docx]

SUPPORTING INFORMATION

**Understanding the relative roles of local environmental, geo-climatic and spatial factors for taxonomic, functional and phylogenetic *β*‐diversity of stream fishes in a large basin, Northeast**

Appendix S1. Summary of local environmental (Local), geo-climatic (Geo) and spatial (Spatial) variables with their codes and descriptions in this study.

| Variables | | |  |  |  |
| --- | --- | --- | --- | --- | --- |
| Code | Unit | Description | Mean | Min | Max |
| **Local** |  | **Local environmental variables** |  |  |  |
| pH | - | pH | 8.55 | 6.73 | 10.12 |
| DO | mg/L | Dissolved oxygen | 9.18 | 0.01 | 15.63 |
| Cond | µs/cm | Conductivity | 249.62 | 3.90 | 1124.00 |
| SS | mg/L | Suspended solid | 37.36 | 1.00 | 1110.00 |
| TDS | mg/L | Total dissolved solid | 201.65 | 14.95 | 903.00 |
| COD | mg/L | Chemical oxygen demand | 14.78 | 0.00 | 84.65 |
| WT | ◦C | Water temperature | 16.29 | 7.20 | 26.00 |
| CODMn | mg/L | Permanganate index of COD | 5.02 | 0.00 | 45.30 |
| NH3-N | mg/L | Ammonia nitrogen | 1.05 | 0.00 | 14.50 |
| NO2-N | mg/L | Nitrite nitrogen | 0.15 | 0.00 | 2.23 |
| PO4-P | mg/L | Soluble reactive phosphorus | 0.07 | 0.00 | 0.93 |
| TN | mg/L | Total Nitrogen | 6.30 | 0.48 | 22.60 |
| TP | mg/L | Total Phosphorus | 0.17 | 0.00 | 2.55 |
| NPR | - | TN/TP | 382.43 | 0.00 | 3986.00 |
| Width | m | Width | 48.78 | 1.50 | 420.00 |
| Velocity | m/s | Velocity | 0.47 | 0.00 | 1.14 |
| Depth | cm | Water depth | 25.96 | 6.33 | 130.00 |
| QHEI | - | Habitat score | 112.38 | 51.00 | 185.00 |
| **Geo** |  | **Geo-climatic variables** |  |  |  |
| Forest | % | Forest% | 67.35 | 5.00 | 100.00 |
| Shrubs | % | Shrubs% | 1.81 | 0.00 | 8.00 |
| Herbaceous | % | Herbaceous vegetation% | 0.34 | 0.00 | 6.00 |
| Agriculture | % | Cultivated and managed vegetation% | 24.17 | 0.00 | 88.00 |
| Urban | % | Urban/built-up% | 0.97 | 0.00 | 17.00 |
| Snow.ice | % | Snow/ice% | 0.36 | 0.00 | 20.00 |
| Barrenlands | % | Barren lands/sparse vegetation% | 4.77 | 0.00 | 19.00 |
| Water | % | Open water% | 0.23 | 0.00 | 1.00 |
| Elevation | m | Elevation of the sampling site | 198.26 | 3.83 | 555.00 |
| Slope | ◦ | Slope of the sampling site | 3.35 | 0.13 | 24.39 |
| Aspect | ◦ | Aspect of the sampling site | 180.57 | 2.07 | 354.81 |
| Bio1 | ◦C | Annual Mean Temperature | 7.44 | 5.19 | 9.00 |
| Bio2 | ◦C | Mean Diurnal Range | 11.51 | 9.72 | 12.75 |
| Bio3 | - | Isothermality | 24.65 | 22.28 | 25.80 |
| Bio4 | - | Temperature Seasonality | 1270.15 | 1200.55 | 1375.42 |
| Bio5 | ◦C | Max Temperature of Warmest Month | 29.26 | 27.50 | 30.50 |
| Bio6 | ◦C | Min Temperature of Coldest Month | -17.44 | -22.00 | -13.30 |
| Bio7 | ◦C | Temperature Annual Range | 46.70 | 43.10 | 51.00 |
| Bio8 | ◦C | Mean Temperature of Wettest Quarter | 22.25 | 20.07 | 23.78 |
| Bio9 | ◦C | Mean Temperature of Driest Quarter | -9.27 | -12.92 | -7.03 |
| Bio10 | ◦C | Mean Temperature of Warmest Quarter | 22.25 | 20.07 | 23.78 |
| Bio11 | ◦C | Mean Temperature of Coldest Quarter | -9.27 | -12.92 | -7.03 |
| Bio12 | mm | Annual Precipitation | 772.15 | 623.00 | 942.00 |
| Bio13 | mm | Precipitation of Wettest Month | 203.54 | 167.00 | 250.00 |
| Bio14 | mm | Precipitation of Driest Month | 6.70 | 4.00 | 10.00 |
| Bio15 | - | Precipitation Seasonality | 103.51 | 98.37 | 108.11 |
| Bio16 | mm | Precipitation of Wettest Quarter | 488.01 | 397.00 | 595.00 |
| Bio17 | mm | Precipitation of Driest Quarter | 24.85 | 16.00 | 35.00 |
| Bio18 | mm | Precipitation of Warmest Quarter | 488.01 | 397.00 | 595.00 |
| Bio19 | mm | Precipitation of Coldest Quarter | 24.85 | 16.00 | 35.00 |
| **Spatial** |  | **Spatial factors** |  |  |  |
| MEM1 | - | Distance-based Moran's eigenvector maps  (MEM1-MEM45) | 0 | -0.962 | 2.298 |
| MEM2 | - |  | 0 | -1.103 | 2.551 |
| … | - |  | … | … | … |
| MEM45 | - |  | 0 | -2.451 | 2.957 |

Variables indicating significant multicollinearity (with variance inflation factor >=3) are excluded. For spatial variables, only MEMs with positive eigenvalues are retained as spatial explanatory variables.

Appendix S2. Species list that observed in this study.

| Species name |
| --- |
| *Abbottina liaoningensis* |
| *Abbottina rivularis* |
| *Acheilognathus chankaensis* |
| *Aristichthys nobilis* |
| *Barbatula nuda* |
| *Carassius auratus* |
| *Carassius auratus gibelio* |
| *Cobitis sibirica* |
| *Cottus poecilopus* |
| *Ctenogobius brunneus* |
| *Ctenogobius cliffordpopei* |
| *Ctenogobius lepturus* |
| *Cyprinius carpio* |
| *Gobio gobio cynocephalus* |
| *Gobio gobio rivuloides* |
| *Gobio lingyuanensis* |
| *Hemiculter leucisculus* |
| *Hemirhamphus sajori* |
| *Huigobio chinssuensis* |
| *Hypomesus olidus* |
| *Hypseleotris swinhonis* |
| *Lampetra morii* |
| *Lefua costata* |
| *Leuciscus waleckii* |
| *Misgurnus anguillicaudatus* |
| *Odontobutis obscura* |
| *Opsariichthys bidens* |
| *Oryzias latipes* |
| *Perccottus glehni* |
| *Phoxinus czekanowskii Dybowski* |
| *Phoxinus lagowskii* |
| *Pseudorasbora parva* |
| *Pungitius sinensis* |
| *Rhinogobius giurinus* |
| *Rhodeus lighti* |
| *Rhodeus sericeus* |
| *Rhodeus sinensis* |
| *Rostrogobio liaoheensis* |
| *Silurus asotus* |
| *Squalidus chankaensis* |
| *Squalidus wolterstorffi* |
| *Zacco platypus* |

https://datadryad.org/stash/share/tYjJIfvvK62LQJOzNNyTSwSP9is7tzw4oQKVwPSC_Po

Appendix S3. Local environmental (*Local*), geo-climatic (*Geo*) and spatial (*Spatial*) factors, based on the results of positive selection of taxonomic *β*-diversity components. The variables are arranged in the order in which they were used in the forward selection procedure. AdjR2Cum (cumulative adjusted R-square), F and p values are shown. All selected variables showed no significant multicollinearity (variance inflation factor < 3). Significance is indicated as * p < 0.05, ** p < 0.01, *** p < 0.001.

| Total | | | | Turnover | | | | Nestedness | | | |
| --- | --- | --- | --- | --- | --- | --- | --- | --- | --- | --- | --- |
| Variables | AdjR^2^Cum | F | p | Variables | AdjR^2^Cum | F | p | Variables | AdjR^2^Cum | F | p |
| *Local**** | | | | *Local**** | | | | *Local** | | | |
| Width | 0.105 | 18.435 | 0.001 | Cond | 0.110 | 19.323 | 0.001 | Cond | 0.046 | 8.244 | 0.001 |
| Cond | 0.167 | 12.047 | 0.001 | Width | 0.168 | 11.454 | 0.001 | Depth | 0.063 | 3.594 | 0.016 |
| Depth | 0.196 | 6.313 | 0.001 | Depth | 0.194 | 5.752 | 0.001 |  |  |  |  |
| COD | 0.219 | 5.366 | 0.001 | Velocity | 0.214 | 4.630 | 0.002 |  |  |  |  |
| Velocity | 0.239 | 4.695 | 0.001 | COD | 0.232 | 4.419 | 0.001 |  |  |  |  |
| PO4 | 0.253 | 3.792 | 0.003 | PO4 | 0.244 | 3.272 | 0.007 |  |  |  |  |
| QHEI | 0.263 | 2.987 | 0.014 | QHEI | 0.253 | 2.804 | 0.013 |  |  |  |  |
| TP | 0.272 | 2.589 | 0.019 | TP | 0.260 | 2.342 | 0.023 |  |  |  |  |
| NH4-N | 0.278 | 2.243 | 0.035 |  |  |  |  |  |  |  |  |
| *Geo**** | | | | *Geo**** | | | | *Geo**** | | | |
| Elevation | 0.151 | 27.408 | 0.001 | Elevation | 0.148 | 26.933 | 0.001 | Bio15 | 0.061 | 10.695 | 0.001 |
| Urban | 0.212 | 12.448 | 0.001 | Urban | 0.201 | 10.788 | 0.001 | Urban | 0.101 | 7.551 | 0.001 |
| Bio15 | 0.247 | 7.847 | 0.001 | Water | 0.231 | 6.681 | 0.001 | Bio14 | 0.128 | 5.637 | 0.003 |
| Bio14 | 0.270 | 5.582 | 0.001 | Bio15 | 0.256 | 5.927 | 0.001 | Barrenlands | 0.154 | 5.414 | 0.005 |
| Water | 0.296 | 6.465 | 0.001 | Bio14 | 0.278 | 5.436 | 0.001 | Water | 0.169 | 3.662 | 0.019 |
| Bio3 | 0.306 | 3.050 | 0.011 | Bio3 | 0.289 | 3.174 | 0.008 |  |  |  |  |
| Snow.ice | 0.312 | 2.225 | 0.043 | Shrubs | 0.299 | 3.040 | 0.006 |  |  |  |  |
| Shrubs | 0.317 | 2.120 | 0.041 | Snow.ice | 0.307 | 2.605 | 0.020 |  |  |  |  |
|  |  |  |  | Herbaceous | 0.312 | 2.076 | 0.045 |  |  |  |  |
| *Spatial**** | | | | *Spatial**** | | | | *Spatial*** | | | |
| MEM6 | 0.084 | 14.622 | 0.001 | MEM6 | 0.072 | 12.604 | 0.001 | MEM8 | 0.046 | 8.144 | 0.001 |
| MEM5 | 0.127 | 8.396 | 0.001 | MEM9 | 0.117 | 8.462 | 0.001 | MEM6 | 0.086 | 7.533 | 0.001 |
| MEM16 | 0.169 | 8.386 | 0.001 | MEM5 | 0.160 | 8.519 | 0.001 | MEM12 | 0.118 | 6.243 | 0.001 |
| MEM9 | 0.211 | 8.685 | 0.001 | MEM16 | 0.200 | 8.412 | 0.001 | MEM5 | 0.129 | 2.905 | 0.040 |
| MEM1 | 0.236 | 5.848 | 0.001 | MEM20 | 0.233 | 7.115 | 0.001 | MEM9 | 0.139 | 2.751 | 0.046 |
| MEM20 | 0.262 | 5.966 | 0.001 | MEM1 | 0.253 | 4.820 | 0.002 | MEM3 | 0.149 | 2.666 | 0.045 |
| MEM4 | 0.278 | 4.322 | 0.001 | MEM4 | 0.271 | 4.548 | 0.002 |  |  |  |  |
| MEM8 | 0.295 | 4.293 | 0.002 | MEM12 | 0.288 | 4.407 | 0.002 |  |  |  |  |
| MEM12 | 0.308 | 3.692 | 0.002 | MEM3 | 0.300 | 3.465 | 0.001 |  |  |  |  |
| MEM3 | 0.317 | 2.836 | 0.013 | MEM8 | 0.311 | 3.142 | 0.008 |  |  |  |  |
| MEM2 | 0.326 | 2.781 | 0.014 | MEM2 | 0.317 | 2.340 | 0.033 |  |  |  |  |
| MEM10 | 0.331 | 2.167 | 0.041 | MEM24 | 0.322 | 2.057 | 0.048 |  |  |  |  |

Bio3= Isothermality, Bio14= Precipitation of Driest Month (mm), Bio15= Precipitation Seasonality.

Appendix S4. Forward selection results for local environment (*Local*), geo-climatic (*Geo*), and spatial (*Spatial*) factors, based on the functional *β*-diversity components. The variables are listed in the order in which they were used in the forward selection procedure. AdjR^2^Cum (cumulative adjusted R^2^), F and p values are shown. All selected variables showed no significant multicollinearity (variance inflation factor < 3). Significance is indicated as * p < 0.05, ** p < 0.01, *** p < 0.001.

| Total | | | | Turnover | | | | Nestedness | | | |
| --- | --- | --- | --- | --- | --- | --- | --- | --- | --- | --- | --- |
| Variables | AdjR^2^Cum | F | p | Variables | AdjR^2^Cum | F | p | Variables | AdjR^2^Cum | F | p |
| *Local**** |  |  |  | *Local**** |  |  |  | *Local** |  |  |  |
| Depth | 0.018 | 3.773 | 0.023 | Width | 0.062 | 10.882 | 0.001 | Cond | 0.043 | 7.755 | 0.001 |
| COD | 0.035 | 3.554 | 0.023 | TP | 0.101 | 7.465 | 0.001 | Depth | 0.066 | 4.570 | 0.004 |
| WT | 0.052 | 3.698 | 0.018 | WT | 0.120 | 4.036 | 0.002 | WT | 0.080 | 3.306 | 0.022 |
|  |  |  |  | Depth | 0.133 | 3.186 | 0.006 | COD | 0.096 | 3.470 | 0.019 |
|  |  |  |  | Cond | 0.141 | 2.463 | 0.023 |  |  |  |  |
| *Geo**** | | | | *Geo**** | | | | *Geo*** | | | |
| Bio15 | 0.063 | 11.097 | 0.001 | Urban | 0.103 | 18.098 | 0.001 | Urban | 0.059 | 10.397 | 0.001 |
| Bio14 | 0.106 | 8.053 | 0.002 | Elevation | 0.132 | 6.009 | 0.001 | Bio15 | 0.095 | 6.851 | 0.002 |
| Urban | 0.142 | 7.070 | 0.001 | Bio15 | 0.165 | 6.820 | 0.001 | Bio14 | 0.117 | 4.616 | 0.005 |
|  |  |  |  | Water | 0.178 | 3.304 | 0.012 |  |  |  |  |
|  |  |  |  | Bio14 | 0.190 | 3.125 | 0.005 |  |  |  |  |
|  |  |  |  | Herbaceous | 0.199 | 2.530 | 0.023 |  |  |  |  |
|  |  |  |  | Bio3 | 0.206 | 2.360 | 0.024 |  |  |  |  |
| *Spatial**** | | | | *Spatial**** | | | | *Spatial*** | | | |
| MEM8 | 0.044 | 7.911 | 0.001 | MEM6 | 0.050 | 8.764 | 0.001 | MEM8 | 0.037 | 6.696 | 0.001 |
| MEM6 | 0.089 | 8.243 | 0.001 | MEM9 | 0.078 | 5.528 | 0.001 | MEM6 | 0.074 | 6.890 | 0.001 |
| MEM5 | 0.115 | 5.248 | 0.005 | MEM12 | 0.105 | 5.523 | 0.001 | MEM16 | 0.097 | 4.845 | 0.002 |
| MEM16 | 0.138 | 5.009 | 0.003 | MEM1 | 0.127 | 4.582 | 0.001 | MEM12 | 0.114 | 3.835 | 0.006 |
| MEM1 | 0.161 | 5.034 | 0.002 | MEM16 | 0.147 | 4.524 | 0.001 | MEM1 | 0.131 | 3.674 | 0.012 |
| MEM17 | 0.176 | 3.473 | 0.014 | MEM8 | 0.165 | 4.053 | 0.004 | MEM9 | 0.146 | 3.605 | 0.013 |
| MEM13 | 0.186 | 2.857 | 0.034 | MEM5 | 0.182 | 3.881 | 0.001 | MEM5 | 0.161 | 3.631 | 0.008 |
| MEM20 | 0.197 | 2.867 | 0.029 | MEM4 | 0.194 | 3.243 | 0.007 | MEM20 | 0.177 | 3.651 | 0.013 |
| MEM3 | 0.207 | 2.747 | 0.038 | MEM20 | 0.207 | 3.234 | 0.008 | MEM3 | 0.191 | 3.487 | 0.017 |
|  |  |  |  | MEM3 | 0.216 | 2.642 | 0.023 | MEM19 | 0.203 | 3.147 | 0.015 |
|  |  |  |  | MEM19 | 0.223 | 2.241 | 0.039 |  |  |  |  |
|  |  |  |  | MEM25 | 0.229 | 2.139 | 0.046 |  |  |  |  |

Bio3= Isothermality, Bio14= Precipitation of Driest Month (mm), Bio15= Precipitation Seasonality.

Appendix S5.Results of forward selection of local environmental (*Local*), geo-climatic (*Geo*) and spatial (*Spatial*) factors for phylogenetic *β*-diversity components respectively. The selected variables are in the order in which they were selected in the forward selection procedure. AdjR^2^Cum (cumulative adjusted R^2^), F and p values are shown. All selected variables show no significant multicollinearity (with variance inflation factor <3). Significance is expressed as * p < 0.05, ** p < 0.01, *** p < 0.001.

| Total | | | | Turnover | | | | Nestedness | | | |
| --- | --- | --- | --- | --- | --- | --- | --- | --- | --- | --- | --- |
| Variables | AdjR^2^Cum | F | p | Variables | AdjR^2^Cum | F | p | Variables | AdjR^2^Cum | F | p |
| *Local**** |  |  |  | *Local**** |  |  |  | *Local* |  |  |  |
| Width | 0.097 | 17.045 | 0.001 | Width | 0.097 | 17.017 | 0.001 | WT | 0.049 | 8.609 | 0.002 |
| Cond | 0.148 | 9.841 | 0.001 | Cond | 0.156 | 11.383 | 0.001 | COD | 0.064 | 3.417 | 0.029 |
| Depth | 0.181 | 6.986 | 0.001 | Depth | 0.178 | 4.972 | 0.002 |  |  |  |  |
| Velocity | 0.200 | 4.299 | 0.001 | Velocity | 0.196 | 4.189 | 0.003 |  |  |  |  |
| COD | 0.215 | 3.809 | 0.003 | COD | 0.205 | 2.619 | 0.018 |  |  |  |  |
| PO4 | 0.226 | 3.137 | 0.010 | PO4 | 0.214 | 2.587 | 0.019 |  |  |  |  |
| TP | 0.234 | 2.494 | 0.018 | QHEI | 0.222 | 2.467 | 0.036 |  |  |  |  |
| NH4-N | 0.242 | 2.365 | 0.034 | NH4-N | 0.227 | 2.014 | 0.049 |  |  |  |  |
| QHEI | 0.248 | 2.200 | 0.036 |  |  |  |  |  |  |  |  |
| *Geo**** | | | | *Geo**** | | | | *Geo**** | | | |
| Elevation | 0.139 | 25.118 | 0.001 | Elevation | 0.138 | 24.764 | 0.001 | Bio15 | 0.068 | 11.938 | 0.002 |
| Urban | 0.197 | 11.657 | 0.001 | Urban | 0.188 | 10.201 | 0.001 | Urban | 0.100 | 6.144 | 0.003 |
| Bio15 | 0.234 | 8.104 | 0.001 | Water | 0.224 | 7.762 | 0.001 | Bio14 | 0.121 | 4.518 | 0.012 |
| Water | 0.256 | 5.340 | 0.002 | Bio15 | 0.241 | 4.407 | 0.001 | Barrenlands | 0.137 | 3.769 | 0.026 |
| Bio14 | 0.277 | 5.082 | 0.001 | Shrubs | 0.257 | 4.049 | 0.001 |  |  |  |  |
| Bio3 | 0.284 | 2.528 | 0.028 | Bio14 | 0.267 | 2.969 | 0.012 |  |  |  |  |
| Shrubs | 0.290 | 2.187 | 0.036 | Bio3 | 0.274 | 2.459 | 0.017 |  |  |  |  |
| *Spatial**** | | | | *Spatial**** | | | | *Spatial*** | | | |
| MEM6 | 0.067 | 11.705 | 0.001 | MEM6 | 0.058 | 10.201 | 0.001 | MEM8 | 0.068 | 11.889 | 0.001 |
| MEM5 | 0.105 | 7.271 | 0.001 | MEM20 | 0.097 | 7.384 | 0.001 | MEM6 | 0.114 | 8.594 | 0.001 |
| MEM16 | 0.143 | 7.467 | 0.001 | MEM9 | 0.135 | 7.503 | 0.001 | MEM12 | 0.146 | 6.631 | 0.003 |
| MEM9 | 0.175 | 6.767 | 0.001 | MEM16 | 0.174 | 7.748 | 0.001 | MEM3 | 0.161 | 3.613 | 0.027 |
| MEM20 | 0.207 | 6.740 | 0.001 | MEM5 | 0.207 | 7.200 | 0.001 | MEM19 | 0.176 | 3.585 | 0.022 |
| MEM1 | 0.234 | 6.095 | 0.001 | MEM1 | 0.226 | 4.415 | 0.003 | MEM10 | 0.188 | 3.121 | 0.029 |
| MEM8 | 0.260 | 6.140 | 0.001 | MEM12 | 0.242 | 4.014 | 0.005 | MEM13 | 0.198 | 2.738 | 0.042 |
| MEM4 | 0.276 | 4.115 | 0.002 | MEM8 | 0.258 | 4.064 | 0.003 | MEM35 | 0.207 | 2.726 | 0.041 |
| MEM3 | 0.287 | 3.061 | 0.009 | MEM4 | 0.273 | 3.870 | 0.003 |  |  |  |  |
| MEM2 | 0.297 | 3.001 | 0.005 | MEM3 | 0.288 | 3.948 | 0.002 |  |  |  |  |
| MEM10 | 0.307 | 2.966 | 0.006 | MEM24 | 0.293 | 2.082 | 0.050 |  |  |  |  |
| MEM12 | 0.315 | 2.738 | 0.017 |  |  |  |  |  |  |  |  |

Bio3= Isothermality, Bio14= Precipitation of Driest Month (mm), Bio15= Precipitation Seasonality.
